# Infant infections, respiratory symptoms, and allergy in relation to timing of rice cereal introduction in a United States cohort

**DOI:** 10.1038/s41598-022-08354-2

**Published:** 2022-03-15

**Authors:** Yuka Moroishi, Antonio J. Signes-Pastor, Zhigang Li, Kathryn L. Cottingham, Brian P. Jackson, Tracy Punshon, Juliette Madan, Kari Nadeau, Jiang Gui, Margaret R. Karagas

**Affiliations:** 1 Department of Epidemiology, Geisel School of Medicine at Dartmouth, Hanover, NH USA; 2 Department of Biomedical Data Science, Geisel School of Medicine at Dartmouth, Hanover, NH USA; 3 Department of Biostatistics, University of Florida, Gainesville, FL USA; 4 Department of Biological Sciences, Dartmouth College, Hanover, NH USA; 5 Children’s Environmental Health and Disease Prevention Research Center at Dartmouth, Hanover, NH USA; 6 Department of Earth Sciences, Dartmouth College, Hanover, NH USA; 7 Department of Pediatrics, Children’s Hospital at Dartmouth, Lebanon, NH USA; 8 Sean N. Parker Center for Allergy and Asthma Research, Stanford University School of Medicine, Stanford, CA USA

**Keywords:** Risk factors, Epidemiology

## Abstract

Rice products marketed in the USA, including baby rice cereal, contain inorganic arsenic, a putative immunotoxin. We sought to determine whether the timing of introduction of rice cereal in the first year of life influences occurrence of infections, respiratory symptoms, and allergy. Among 572 infants from the New Hampshire Birth Cohort Study, we used generalized estimating equation, adjusted for maternal smoking during pregnancy, marital status, education attainment, pre-pregnancy body mass index, maternal age at enrollment, infant birth weight, and breastfeeding history. Among 572 infants, each month earlier of introduction to rice cereal was associated with increased risks of subsequent upper respiratory tract infections (relative risk, RR = 1.04; 95% CI: 1.00–1.09); lower respiratory tract infections (RR = 1.19; 95% CI: 1.02–1.39); acute respiratory symptoms including wheeze, difficulty breathing, and cough (RR = 1.10; 95% CI: 1.00–1.22); fever requiring a prescription medicine (RR = 1.22; 95% CI: 1.02–1.45) and allergy diagnosed by a physician (RR = 1.20; 95% CI: 1.06–1.36). No clear associations were observed with gastrointestinal symptoms. Our findings suggest that introduction of rice cereal earlier may influence infants’ susceptibility to respiratory infections and allergy.

## Introduction

Early life is a critical period of immune system development and impacts health lifelong^1,2^. Infections remain the leading causes of mortality in children under five years of age around the world^3^. Feeding practices, in particular breast feeding, are known to protect against infections and improve child health outcomes^4^; however, far less is known about the impact of diet during infants’ transition to solid foods. Rice is an important dietary source of arsenic, including rice products commonly fed to infants as a first food and as snacks^5–8^. In flooded rice paddy fields, rice grains accumulate arsenic at rates about 10 times higher than that of other grains^6,9,10^. Additionally, arsenicals such as monosodium methanearsonate and disodium methanearsonate were used in pesticides and herbicides. Although these compounds are now mostly banned, residues remain in soil^9,11,12^. While rice cereal fortified with iron may be a good source of nutrients, concerns have been raised about this practice because of the arsenic content of rice-based products^13^. In previous studies from our cohort, infant urinary arsenic concentrations increased with consumption of rice products during infants transition to solid food^14^, and at one year of age, infants fed rice products had elevated urinary concentrations of arsenic compared to those who were not fed these products^15^.

Exposure to arsenic early in life has been specifically associated with an impaired immune response and increased risk of infection^16–18^. Infants are especially vulnerable to respiratory infections, in part due to their immature immune system^19^. Studies have reported associations between in utero arsenic exposure and a number of adverse outcomes including infant infections and respiratory outcomes among highly exposed populations in Bangladesh and among US infants^20–22^. These findings are supported by mechanistic evidence that in utero arsenic exposure may influence the epigenome of the placenta^23^, immune cell profiles in newborn cord blood^24^, and the infant gut microbiome^25^. While epidemiologic data are lacking on allergy outcomes, maternal urinary arsenic concentrations during pregnancy were related to higher activated Th2 cells, which produce cytokines responsible for IgE production, a marker of allergic response^26–29^. In addition, there is evidence that early arsenic exposure influences childhood infections risk in highly exposed populations^30,31^.

Despite health concerns^32,33^, a regulatory limit for arsenic in infant rice cereal has not yet ratified in the USA. The European Union (EU) established a standard for inorganic arsenic in infant rice products to a maximum level of 100 μg/kg^34^. The US Food and Drug Administration (FDA) proposed the same guidance for infant rice cereal in 2016^35^. In 2018, the Governmental Accountability Office recommended that the FDA and US Department of Agriculture coordinate their efforts to identify contaminants in food including arsenic and establish a timeline for finalizing the guidance^36^. An action level has been set by the FDA for apple juice, but not other foods^37^. In 2006, the USA set the maximum contaminant level for inorganic arsenic in drinking water to be 10 μg/L^38^, but evidence on the detrimental health impacts at even lower levels of exposure led to the reduction of the drinking water standard in certain states, including New Jersey^39^ and New Hampshire^40^, to 5 μg/L. In light of the vulnerability of infants to early life environmental exposures, we investigated the timing of introduction of rice cereal during their transition to solid food in first year of life and subsequent risk of infections, immune-related symptoms, and allergies as part of the New Hampshire Birth Cohort Study (NHBCS).

## Results

### Baseline characteristics

Of the 1760 pregnancies enrolled in the NHBCS as of October 2017, a total of 983 infants had complete follow-up data up to at least age of 8 months. After removing missing values on rice cereal consumption (54 missing) and susbsequent health outcome information (357 missing), the final dataset contained 572 infants (Supplementary Fig. S1). We found that the characteristics of the 411 subjects excluded from our analyses were generally similar to that of included subjects, with the exception of marital status (Supplementary Table S1). Our study group included a roughly equal distribution of male (54%) and female (46%) infants (Table 1). Among infants who were introduced to rice cereal in the first year of life, the average age at introduction was 5.2 months (SD: 1.3 months) (Supplementary Table S2). At the 4 month, 8 month, and 12 month time periods, rice cereal was consumed in 11.7%, 69.6%, and 68.6% of infants respectively (Supplementary Table S2). Overall, 96.5% of infants were reporting as having at least one infection or symptom of any duration reported up to age 18 months, 91.4% having at least one lasting 2 or more days, 65.2% having at least one involving a doctor’s visit, and 52.3% having at least one resulting in a prescription medication (Supplementary Table S2). For allergies, 13.5% of infants were reporting as having at least one allergy, and 7.9% having at least one diagnosed by a doctor (Supplementary Table S2). Sample sizes and proportions of each outcome for each follow-up interval are reported in Supplementary Table S3. Household tap water arsenic concentrations were generally low, with a mean 2.2 μg/L (SD: 7.1; range: 0.0 to 92.3), but with 11.1% of the study population having levels above the New Hampshire drinking water standard of 5 μg/L (Table 1).

**Table 1:** Selected characteristics of mothers and infants (N = 572) in the new hampshire birth cohort study followed to age 18 months^a^.

Variable	Sample size	Mean (SD) or No. (%)
**Maternal characteristics**		
Smoking during any trimester of pregnancy, No. (%)	552	
Yes		61 (11.1)
No		491 (88.9)
Relationship status, No. (%)	538	
Married		486 (90.3)
Single		43 (8.0)
Separated/divorced		9 (1.7)
Highest level of educational attainment, No. (%)	537	
≤ High school/GED		52 (9.7)^b^
Some college		90 (16.8)^b^
College graduate		214 (39.9)^b^
Postgraduate schooling		181(33.7)^b^
BMI before pregnancy (kg/m^2^), mean (SD)	560	26.1 (5.7)
Age at enrollment (years), mean (SD)	572	31.9 (4.8)
Arsenic in water (μg/L), mean (SD)	552	2.2 (7.1)
Water Arsenic > 5 μg/L, No. (%)	552	61 (11.1)
**Infant characteristics**		
Sex, No. (%)	572	
Male		310 (54.2)
Female		262 (45.8)
Birth weight (g), mean (SD)	555	3416.7 (522.8)
Ever breast fed at 4 months, No. (%)	537	
Yes		513 (95.5)
No		24 (4.5)
Other solid food consumption at 4 months, No. (%)^c^	358	
Yes		14 (3.9)
No		344 (96.1)
Other solid food consumption at 8 months, No. (%)^c^	437	
Yes		120 (27.5)
No		317 (72.5)
Other solid food consumption at 12 months, No. (%)^c^	544	
Yes		137 (25.2)
No		407 (74.8)

*SD* standard deviation, *No*. number, *BMI* body mass index.

^a^Of the 572 participants included in the analyses, 482 participants (84.3%) had data at least one time-period of rice cereal consumption, health outcome data for a subsequent time period, and complete data on confounders. Smoking during pregnancy was missing for 20 (3.50%) mothers, relationship status was missing for 34 (5.94%) mothers, education was missing for 35 (6.12%) mothers, and pre-pregnancy BMI was missing for 12 (2.10%) mothers. Birth weight was missing for 17 (2.97%) and breast-feeding status was missing for 59 (10.31%) infants. Other solid food consumption at 8 months was missing for 1 (0.23%) infant. A total of 20 (3.50%) participants had missing data for arsenic species in water.

^b^Percentages do not sum to 100 due to rounding.

^c^Percentage calculated using different sample sizes due to missing values. Sample sizes were 321, 373, and 464 for 4 months, 8 months, and 12 months respectively.

### Rice cereal and infections, respiratory symptoms, and allergy

**Figure 1:**
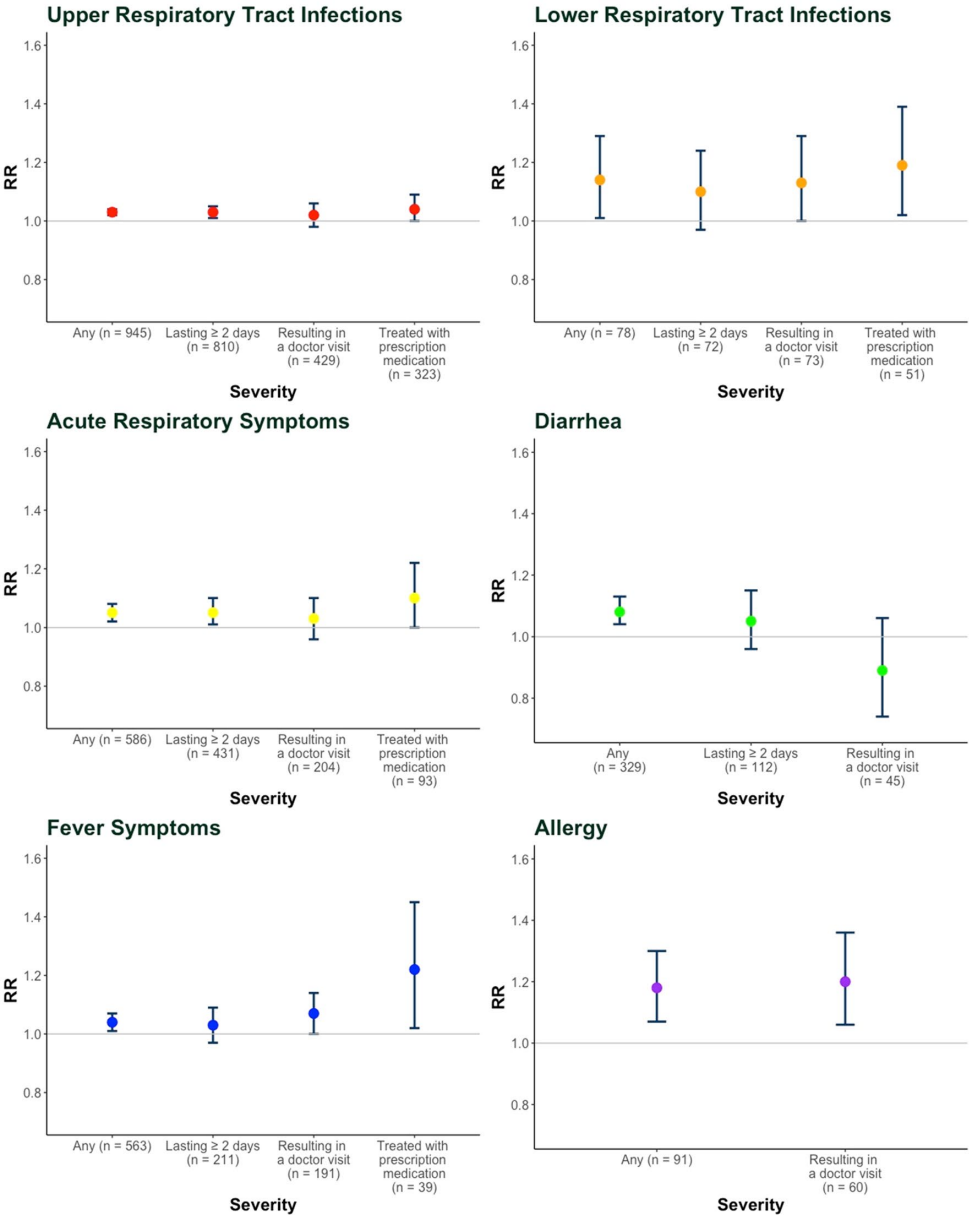
Adjusted Risk Ratios (95% CIs) for Each Month Earlier of Introduction of Rice Cereal According to Outcome Severity, N = 572 infants. RR indicates risk ratio for health outcomes according to reported severity. The circles indicate RR at each severity level for each outcome. The lines indicate the 95% confidence intervals. The grey line represents the null RR = 1. Number of total outcome cases in three repeated measures is denoted by n. Number of observed outcomes can be higher due to repeated events among time points for each infant. Abbreviations: *RR* risk ratio. Sample size N = 571 for fever analyses.

In our GEE analysis, earlier introduction of rice cereal was associated with increased risks of lower respiratory tract infections (i.e. bronchitis, pneumonia, bronchiolitis, whooping cough, and respiratory syncytial virus), respiratory symptoms, fever, and allergies, and to a lesser extent upper respiratory tract infections (i.e. runny stuffed nose, eye infection, ear infection, severe flu, sinus infection, strep throat, and laryngitis), but not gastrointestinal symptoms (Fig. 1). While the magnitudes of the associations did not differ greatly across the variables for a given outcome and the confidence intervals overlapped, there was a tendency for the risk ratios to be higher for outcomes involving a health care provider visit or a medication prescribed (Fig. 1, Supplementary Table S4). Relative risk estimates for upper and lower respiratory tract infections requiring a prescription medicine increased by 4% (RR = 1.04; 95% CI: 1.00–1.09) and by 19% (RR = 1.19; 95% CI: 1.02–1.39) for each month earlier that rice cereal was introduced. A 10% increase in the relative risk of acute respiratory symptoms requiring a prescription medicine (RR = 1.10; 95% CI: 1.00–1.22) and 22% increase increase of fever symptoms requiring prescription medicine (RR = 1.22; 95% CI: 1.02–1.45) were observed for each month earlier that rice cereal was introduced. For reported allergies diagnosed by a doctor, the relative risk estimate was 20% higher for each month earlier that rice cereal was introduced (RR = 1.20; 95% CI: 1.06–1.36), and for this outcome, the relative risk estimate was similar to any reported allergy. We did not observe any consistent associations with diarrhea, including symptoms requiring a doctor’s visit (RR = 0.89; CI, 0.74–1.06). Only three cases of diarrhea had a medication prescribed, so this outcome was not included in the GEE analysis. Risk ratio estimates and confidence intervals for covariates included in our models are provided in Supplementary Table S5. Results for crude analyses are provided in Supplementary Table S6.

## Discussion

In our prospective cohort study, we found that earlier introduction of rice cereal to an infant’s diet was associated with higher risks of both upper and lower respiratory tract infections, respiratory symptoms, fever, and allergies. These associations were slightly stronger for what may have been more severe outcomes of lower respiratory tract infection, respiratory symptoms, and fever, i.e., those characterized by having a medication prescribed.

Infections remain the most important cause of morbidity in young children, and allergic and atopy diseases are becoming more widespread^3^. In the USA, an estimated 42.8% of infant hospitalizations in 2003 were due to infections^41^. Of these, 59.0% were due to lower respiratory tract infections and 6.5% to upper respiratory tract infections^41^. In 2013, the Centers for Disease Control and Prevention (CDC) noted an increasing trend in childhood food and skin allergies from 1997 to 2011^42^. In a 2017 CDC survey, 13% of children under the age of 18 years had been told they had asthma, 11% a respiratory allergy, 6.5% a food allergy, and 13.5% a skin allergy^43^. Thus, efforts to reduce infection and allergy prevalence in infants is of critical public health importance.

Early feeding practices play a critical role in the developing immune system of infants^4^, but there are limited studies of the impact of infants’ transition to solid foods. In a large prospective study from Dundee, Scotland (N = 545), early introduction of solid food was related to an increased risk of infant wheeze, but not other respiratory illnesses^44^. One prospective cohort study from the United Kingdom found infant introduction to solid foods before 4 months of age was associated with higher odds of any diarrhea compared to those introduced after 4 months of age (N = 615)^45^. Introduction of wheat after 6 months of age compared to before or equal to 6 months of age was associated with a reduced risk of wheat allergies in a longitudinal birth cohort from Denver (N = 1612)^46^. While research linking infant rice cereal exposure to later health outcomes is lacking, our results align with previous studies that observed increased risks of infection with arsenic exposure in early childhood. A Bangladeshi cohort study of children aged 7–17 years who were exposed to high levels of arsenic in utero and early childhood from contaminated drinking water and sex- and age-matched controls without such exposures observed increased respiratory symptoms such as wheezing and shortness of breath (N = 650) in the high arsenic exposure group^30^. In a separate case control study from Bangladesh of children aged 28 days to 59 months who were hospitalized with severe and very severe pneumonia and age-matched controls, the odds of pneumonia were elevated among children with higher urinary arsenic concentrations measured both during hospitalization and at the convalescent period (30 days after) (N = 449)^31^. Other cohort studies from Bangladesh and from the USA have also associated childhood infections and diarrhea in relation to in utero arsenic exposure^20–22^.

Rice products are a well-recognized route of exposure to arsenic. Arsenic exposure has been shown to increase risk of infections and other diseases, and emerging evidence points to the toxicological effects of arsenic on immune function^16,47^. The World Health Organization established a guideline for arsenic in drinking water of 10 μg/L in 1993 and had acknowledged arsenic contamination in rice and rice products as a public health concern^48,49^. In a 2014 EU report, concentrations of inorganic arsenic ranged from 56 to 268 μg/kg for infant rice products^50^. A similar report from the USA covering 2012 to 2016 reported inorganic arsenic concentrations ranging from 21 to 151 μg/kg for infant white rice cereal and from 30 to 254 μg/kg for infant brown rice cereal^51^. In our cohort, 80% of infants were introduced to rice cereal in the first year of life, and more than half of our infants were eating rice products at one year of age^15^. Further, urinary arsenic concentrations increased with the number of rice and rice product servings^15^. The American Academy of Pediatrics has raised awareness about arsenic exposure from feeding infant rice products and recommends feeding infants a variety of foods with a variety of textures^8,52^.

Our study has a number of strengths, but also has limitations that need to be noted. Our study benefitted from the availability of prospective cohort data of carefully collected repeated measurements of infection occurrences, respiratory symptoms, diarrhea, and allergies; timing of introduction of rice cereal; and a broad range of potential confounding factors. Among our main limitations was our inability to quantify the concentrations of arsenic to which infants were exposed through rice cereal. This is in part because the concentrations of arsenic in rice depends on a number of factors including genotype, cultivation, and irrigation techniques, and concentration can be altered by cooking techniques^53,54^. Despite the heterogeneity of arsenic concentrations in rice, we previously found rice cereal to be a contributor to arsenic exposure among our infants^14^. In our analyses, we also adjusted for household water arsenic concentration along with indicator variables for breastfeeding and consumption of other solid foods. Participant recall is a potential source of bias. Efforts to minimize non-differential misclassification were made by including questions on duration of the illness and asking whether the infant saw a doctor or was prescribed medicine for their condition. Stronger associations were found with outcomes involving medical care, which would be expected to have higher validity and reflect greater severity of illness. Furthermore, self-reporting of allergies that were not medically confirmed was another potential source of misclassification. We have found that responses from caregiver responses for infections and symptoms that involved a doctor visit in our cohort tend to be at least 80% concordant with their pediatric medical records (unpublished data). Our findings of an increased risk of any reported diarrhea associated with earlier introduction rice consumption should thus be interpreted with caution as the relative risk estimates were not consistently elevated for diarrhea lasting 2 or more days or associated with a doctor’s visit. The possibility of unmeasured confounding also cannot be excluded; however, we assessed the potential confounding of factors previously found to be related to our outcomes of interest and found that certain factors such as family history of allergy, day care attendance, and parity were not associated with timing of rice cereal introduction. The price of rice cereal in our cohort may be indicative of socio-economic status. Although we did control for maternal marital status and highest level of education attainment, residual confounding remains a possibility. We inspected the costs of infant cereals from five major grocery stores in a region of the state where recruitment took place. We did not find that the costs of infant rice-based cereal products differed appreciably from that of other grain cereals. Lastly, we computed 95% confidence intervals for our analyses, which do not account for multiple comparisons.

In conclusion, our findings suggest that earlier introduction of rice cereal may increase an infant’s risk of infections, respiratory symptoms, and allergies. The widespread occurrence of these outcomes in young children and use of rice cereal as a first food underscores the importance of considering the types and timing of foods introduced when providing dietary recommendations for infants.

## Methods

### Study design

Participants in this study include mother-infant dyads from the NHBCS. Pregnant women aged 18 to 45, receiving prenatal care at study clinics in New Hampshire, USA, were recruited starting in January 2009 as described previously^55,56^. The cohort includes only women who were living in the same household since their last menstrual period, not planning to move, living in a household served by a private water system, and with a singleton pregnancy. Participants completed surveys, including questions on sociodemographic factors, lifestyle such as smoking history, and pre-pregnancy body mass index (BMI), and infant birth characteristics were ascertained from a review of the delivery medical records. Home tap water samples were collected and analyzed by inductively coupled mass spectrometry to detect arsenic species^57^. The Committee for the Protection of Human Subjects at Dartmouth College approved all protocols, and participants provided written informed consent upon enrollment. All methods were performed in accordance with relevant guidelines and regulations.

### Data collection

Telephone interviews were conducted with caregivers when the infants turned 4, 8, and 12 months of age and at 6-month intervals thereafter. The survey asked whether or not their infant ever consumed rice cereal from birth and the day of the telephone interview (yes/no) and the month (or age in months) that rice cereal was introduced to their diet. Caregivers were asked whether their child had any infections, acute respiratory symptoms (e.g., wheeze, difficulty breathing, and cough), diarrhea or fever since the last time they were interviewed. For positive responses, participants were asked to report if the condition lasted for more than 2 days, if the child saw a doctor, and if the child received prescription medicine for the condition. Participants were further asked whether their child had any known allergy (e.g., cats or dogs, antibiotics, dust, grass and plants, pollen, insect bites, peanuts, other nuts, eggs, and other foods), and for positive responses, if the allergy was diagnosed by a doctor.

### Statistical analysis

We examined rice cereal intake prior to the occurrence of infection. Specifically, we examined months since first introduction of rice cereal by 4 months of age on 8-month outcomes, months since first introduction of rice cereal by 8 months of age on 12-month outcomes, and months since first introduction of rice cereal by 12 months of age on 18-month outcomes. For example, we examined number of months since rice cereal consumption of a subject at 8 months, on occurrence of health outcome at 12 months, which is the subsequent survey collection interval. For each interval (i.e., 4, 8, or 12 months), we computed the number of months since rice cereal was introduced and included an indicator variable of whether rice cereal was consumed in that interval (yes/no). We multiplied this indicator variable by number of months since rice cereal was introduced as our main predictor.

We then used Generalized Estimating Equation (GEE) with Poisson regression with AR(1) correlation structure and robust variance to assess the association between months since introduction of rice cereal exposures and repeated measures of longitudinal outcomes^58^. Factors associated with both rice exposure, as determined from our data (Supplementary Table S7), and outcomes, considered a priori and used in previous studies^22^, were considered potential confounders and included in our models. These included smoking during pregnancy (yes/no), maternal relationship status (married, single, separated/divorced), maternal education (≤ high school/GED, some college, college graduate, postgraduate schooling), maternal pre-pregnancy BMI, maternal age of enrollment (years), infant birth weight (grams), breastfeeding status as of four months (ever/never), and consumption of other solid food than rice cereal (ever/never) at each time point. Because water can be used to make rice cereal and is also a surrogate for in utero arsenic exposure, we also adjusted for total arsenic concentrations measured in household tap water samples (μg/L) in our analyses. For interpretability, we exponentiated the coefficient values to obtain relative risk (RR) and 95% confidence intervals (CI). Of the 572 participants included in the analyses, 482 participants (84.3%) had complete data for at least one time interval on rice cereal consumption and on subsequent health outcomes along with all potentially confounding variables considered. For the other 90 participants, we assumed for the values for potential confounders were missing at random using multiple imputation by chained equations and the predictive mean matching method to impute missing data^59^. All analyses were performed using R version 3.4.3 and functions *mice* and *geeglm* in packages ‘mice’ and ‘geepack’.

## Supplementary Information

Supplementary Information.
