# Supplementary material for: Infant infections, respiratory symptoms, and allergy in relation to timing of rice cereal introduction in a United States cohort
Source: Sci Rep. 2022 Mar 15;12:4450. doi: 10.1038/s41598-022-08354-2 (PMC8924265; doi:10.1038/s41598-022-08354-2)
Supplement: Supplementary file 1 — Supplementary Information. [file 41598_2022_8354_MOESM1_ESM.docx]

**Supplementary Information:**

**Infant Infections, Respiratory Symptoms, and Allergy in Relation to Timing of Rice Cereal Introduction in a United States Cohort**

Yuka Moroishi^1,2^, Antonio J. Signes-Pastor^1^, Zhigang Li^3^, Kathryn L. Cottingham^4,5^, Brian P. Jackson^6^, Tracy Punshon^4,5^, Juliette Madan^1,5,7^, Kari Nadeau^8^, Jiang Gui^2^, Margaret R. Karagas^1,5*^.

^1^Department of Epidemiology, Geisel School of Medicine at Dartmouth, Hanover, New Hampshire, USA.

^2^Department of Biomedical Data Science, Geisel School of Medicine at Dartmouth, Lebanon, New Hampshire, USA.

^3^Department of Biostatistics, University of Florida, Gainesville, Florida, USA.

^4^Department of Biological Sciences, Dartmouth College, Hanover, New Hampshire, USA.

^5^Children’s Environmental Health and Disease Prevention Research Center at Dartmouth, Hanover, New Hampshire, USA.

^6^Department of Earth Sciences, Dartmouth College, Hanover, New Hampshire, USA.

^7^Department of Pediatrics, Children's Hospital at Dartmouth, Lebanon, New Hampshire, USA.

^8^Sean N. Parker Center for Allergy and Asthma Research, Stanford University School of Medicine, Stanford, California, USA.

*Corresponding author’s email: Margaret.R.Karagas@dartmouth.edu

Supplementary Figure S1: Flow Diagram of Study Participants with Inclusion and Exclusion Criteria

**1760** NHBCS pregnancies enrolled as of October 2017

**1760** Infants with at least one complete follow-up survey

**808** Excluded due to incomplete follow up to age of 8 months

**983** Infants with follow-up data from 8 to 18 months

**54** excluded due to restriction to those with rice cereal consumption information, and **357** excluded due to additional restriction to those with incomplete outcome data at the next follow-up interval

**572** Infants included in GEE analysis

Abbreviations: *NHBCS* New Hampshire Birth Cohort Study, *GEE* generalized estimating equation.

Supplementary Table S1. Selected Characteristics of Mothers and Infants (N = 411) in the New Hampshire Birth Cohort Study Not Included in Main Analyses Followed to Age 18 Months

| Variable | Sample Size | Mean (SD) or No. (%) |
| --- | --- | --- |
| **Maternal Characteristics** | | |
| Smoking during any trimester of pregnancy, No. (%) | 372 |  |
| Yes |  | 55 (14.8%) |
| No |  | 317 (85.2 %) |
| Relationship status, No. (%) | 366 |  |
| Married |  | 293 (80.1) |
| Single |  | 56 (15.3) |
| Separated/Divorced |  | 17 (4.6) |
| Highest level of educational attainment, No. (%) | 367 |  |
| ≤high school/GED |  | 49 (13.4)^a^ |
| Some college |  | 70 (19.1)^a^ |
| College graduate |  | 135 (36.8) ^a^ |
| Postgraduate schooling |  | 113 (30.8)^a^ |
| BMI before pregnancy (kg/m^2^), mean (SD) | 398 | 26 (6.0) |
| Age at enrollment (years), mean (SD) | 411 | 31.1 (4.9) |
| Arsenic in water (μg/L), mean (SD) | 390 | 4.2 (13.5) |
| Water Arsenic > 5 μg/L, No. (%) | 390 | 66 (16.9) |
| **Infant Characteristics** | | |
| Sex, No. (%) | 410 |  |
| Male |  | 188 (45.9) |
| Female |  | 222 (54.1) |
| Birth weight (g), mean (SD) | 398 | 3447 (496) |
| Ever breast fed at 4 months, No. (%) | 350 |  |
| Yes |  | 333 (95.1) |
| No |  | 17 (4.9) |
| Other solid food consumption at 4 months, No. (%) ^c^ | 88 |  |
| Yes |  | 78 (88.6) |
| No |  | 10 (11.4) |
| Other solid food consumption at 8 months, No. (%) ^c^ | 47 |  |
| Yes |  | 26 (55.3) |
| No |  | 21 (44.7) |
| Other solid food consumption at 12 months, No. (%) ^c^ | 315 |  |
| Yes |  | 53 (16.8) |
| No |  | 262 (83.2) |

^a^ Percentages do not sum to 100 due to rounding

Supplementary Table S2: Infant Rice Cereal Consumption and Immune-Related Outcome Prevalence

| Variable | N | Mean (SD)  or N (%) |
| --- | --- | --- |
| Ever consumed rice cereal before age of 12 months | 572 |  |
| Yes |  | 435 (76.0) |
| No |  | 137 (24.0) |
| Age of rice cereal introduction of those consumed before age of 12 months (months) | 435 | 5.2 (1.3) |
| Rice cereal introduced before 4 months of age |  | 27 (6.2) |
| Rice cereal introduced between 4-8 months of age |  | 381 (87.6) |
| Rice cereal introduced between 8-12 months of age |  | 27 (6.2) |
| Rice cereal consumption at 4 months^a^ | 358 |  |
| Yes |  | 42 (11.7) |
| No |  | 316 (88.3) |
| Time since rice cereal introduction at 4 months (months) | 435 | 0.02 (0.1) |
| Rice cereal consumption at 8 months^a^ | 438 |  |
| Yes |  | 305 (69.6) |
| No |  | 133 (30.4) |
| Time since rice cereal introduction at 8 months (months) | 572 | 1.4 (1.3) |
| Rice cereal consumption at 12 months^a^ | 544 |  |
| Yes |  | 373 (68.6) |
| No |  | 171 (31.4) |
| Time since rice cereal introduction at 12 months (months) | 572 | 4.4 (2.7) |
| Infections or symptoms within 5-18 months of life | 572 |  |
| At least one outcome |  | 552 (96.5) |
| At least one outcome lasting ≥ 2 days |  | 523 (91.4) |
| At least one outcome resulting in a doctor visit |  | 373 (65.2) |
| At least one outcome treated with prescription medication |  | 299 (52.3) |
| Allergy within 5-18 months of life | 572 |  |
| At least one outcome |  | 77 (13.5) |
| At least one outcome resulting in a doctor visit |  | 45 (7.9) |
| Reported an allergy to peanuts |  | 5 (0.9) |
| Reported an allergy to other nuts |  | 3 (0.5) |
| Reported an allergy to eggs |  | 7 (1.2) |
| Reported an allergy to other foods |  | 39 (6.8) |
| Reported an allergy to antibiotics |  | 21 (3.7) |
| Reported an allergy to cats or dogs |  | 4 (0.7) |
| Reported an allergy to pollen |  | 9 (1.6) |
| Reported an allergy to latex |  | 2 (0.3) |
| Reported an allergy to dust |  | 2 (0.3) |
| Reported an allergy to insect bites |  | 2 (0.3) |
| Reported an allergy to grass |  | 5 (0.9) |

Abbreviations: *SD* standard deviation.

^a^Percentage calculated using different sample sizes due to missing values. Sample sizes were 358, 438, and 544 for 4 months, 8 months, and 12 months respectively.

Supplementary Table S3. Number of Immune-related Outcomes over 5-18 Months at Each Time Period, N = 572

| Outcome | Time Period | Any report of the outcome  No.(%) | Lasting ≥ 2 days  No.(%) | Involving a doctor visit  No.(%) | Requiring a prescription medication  No.(%) |
| --- | --- | --- | --- | --- | --- |
| Upper Respiratory Infections | 8 months | 206 (74.9) | 177 (64.4) | 103 (37.5) | 58 (21.1) |
|  | 12 months | 340 (83.1) | 292 (71.4) | 155 (37.9) | 119 (29.1) |
|  | 18 months | 399 (90.9) | 341 (77.7) | 171 (38.9) | 146 (33.3) |
| Lower Respiratory Infections | 8 months | 17 (6.2) | 17 (6.2) | 16 (5.8) | 9 (3.3) |
|  | 12 months | 28 (6.8) | 27 (6.6) | 26 (6.4) | 18 (4.4) |
|  | 18 months | 33 (7.5) | 28 (6.4) | 31 (7.1) | 24 (5.5) |
| Acute Respiratory Symptoms | 8 months | 133 (48.4) | 99 (36.0) | 53 (19.3) | 18 (6.5) |
|  | 12 months | 202 (49.4) | 143 (35.0) | 70 (17.1) | 31 (7.6) |
|  | 18 months | 251 (57.2) | 189 (43.1) | 81 (18.5) | 44 (10.0) |
| Diarrhea | 8 months | 46 (16.7) | 15 (5.5) | 11 (4.0) | 0 (0.1) |
|  | 12 months | 124 (30.3) | 40 (9.8) | 16 (3.9) | 2 (0.5) |
|  | 18 months | 159 (36.2) | 57 (13.0) | 18 (4.1) | 1 (0.2) |
| Fever Symptoms ^a^ | 8 months | 101 (36.7) | 32 (11.6) | 36 (13.1) | 4 (1.5) |
|  | 12 months | 210 (51.3) | 84 (20.5) | 70 (17.1) | 12 (2.9) |
|  | 18 months | 252 (57.8) | 95 (21.6) | 85 (19.4) | 23 (5.2) |
| Allergy | 8 months | 11 (4.0) | N/A^b^ | 6 (2.2) | N/A^b^ |
|  | 12 months | 27 (6.6) |  | 17 (4.2) |  |
|  | 18 months | 53 (12.1) |  | 37 (8.4) |  |

^a^ Sample size N = 571 for fever analyses

^b^ Participants only asked about any allergies and whether these allergies had been doctor diagnosed

Supplementary Table S4. Adjusted Risk Ratio Estimates and 95% Confidence Intervals From GEE in Repeated Measures over 5-18 Months for One Month Earlier Introduction of Rice Cereal on Risk of Immune-related Outcomes, N = 572^a^.

| Outcome | Any report of the outcome  RR (95% CI)  N of total events | Lasting ≥ 2 days  RR (95% CI)  N of total events | Involving a doctor visit  RR (95% CI)  N of total events | Requiring a prescription medication  RR (95% CI)  N of total events |
| --- | --- | --- | --- | --- |
| Upper Respiratory Infections | 1.03  (1.02-1.04)  945 | 1.03  (1.01-1.05)  810 | 1.02  (0.98-1.06)  429 | 1.04  (1.00-1.09)  323 |
| Lower Respiratory Infections | 1.14  (1.01-1.29)  78 | 1.10  (0.97-1.24)  72 | 1.13  (1.00-1.29)  73 | 1.19  (1.02-1.39)  51 |
| Acute Respiratory Symptoms | 1.05  (1.02-1.08)  586 | 1.05  (1.01-1.10)  431 | 1.03  (0.96-1.10)  204 | 1.10  (1.00-1.22)  93 |
| Diarrhea | 1.08  (1.04-1.13)  329 | 1.05  (0.96-1.15)  112 | 0.89  (0.74-1.06)  45 | ^c^  3 |
| Fever Symptoms ^b^ | 1.04  (1.01-1.07)  563 | 1.03  (0.97-1.09)  211 | 1.07  (1.00-1.14)  191 | 1.22  (1.02-1.45)  39 |
| Allergy | 1.18  (1.07-1.30)  91 | N/A ^d^ | 1.20  (1.06-1.36)  60 | N/A^d^ |

Abbreviations: RR, risk ratio; CI, confidence interval.

^a^ GEE adjusted for smoking during pregnancy, maternal relationship status, maternal education, maternal pre-pregnancy BMI, maternal age of enrollment, arsenic concentrations in household tap water samples, infant birth weight, breastfeeding, and other solid food consumption. Risk ratios represent increased risk of health outcome with every month earlier of introduction to rice cereal.

^b^ Sample size N = 571 for fever analyses

^c^ Too few observations to perform analysis

^d^ Participants only asked about any allergies and whether these allergies had been doctor diagnosed

Supplementary Table S5. Crude Risk Ratio Estimates and 95% Confidence Intervals From GEE for all Other Covariates on Risk of Immune-related Outcomes, N = 572^a^.

| Variable | Upper Respiratory Infections | Lower Respiratory Infections | Acute Respiratory Symptoms | Diarrhea | Fever Symptoms^b^ | Allergy |
| --- | --- | --- | --- | --- | --- | --- |
| Intercept | 0.21  (0.06, 0.66) | 0.00  (0.00, 0.07) | 0.02  (0.00, 0.20) | 0.14  (0.00, 5.82) | 0.01  (0.00, 0.26) | 5.98  (0.17, 212.93) |
| Indicator of Rice Cereal Consumption | 1.32  (0.98, 1.78) | 0.70  (0.27, 1.83) | 0.77  (0.41, 1.43) | 2.35  (0.82, 6.72) | 0.94  (0.24, 3.62) | 1.42  (0.53, 3.84) |
| Other Solid Food Consumption | 1.19  (0.89, 1.63) | 1.07  (0.44, 2.57) | 1.43  (0.83, 2.48) | 2.22  ()0.86, 5.78 | 2.04  (0.66, 6.29) | 2.68  (1.02, 7.05) |
| Smoking during any trimester of pregnancy | 1.26  (0.91, 1.74) | 0.80  (0.29, 2.18) | 1.35  (0.67, 2.71) | 1.36  (0.63, 2.94) | 0.89  (0.35, 2.30) | 0.56  (0.20, 1.59) |
| Relationship status – Married (baseline) VS Single | 1.01  (0.65, 1.58) | 0.95  (0.32, 2.80) | 2.09  (1.01, 4.32) | 1.91  (0.70, 5.19) | 0.73  (0.19, 2.91) | 0.89  (0.30, 2.63) |
| Relationship status – Married (baseline) VS Separated/Divorced | 1.28  (0.76, 2.16) | 0.86  (0.11, 6.58) | 2.26  (0.83, 6.18) | 1.41  (0.27, 7.39) | 3.05  (0.87, 10.71) | 1.90  (0.20, 17.77) |
| Highest level of educational attainment – ≤high school/GED (baseline) VS Some college | 0.78  (0.50, 1.22) | 0.82  (0.28, 2.39) | 1.04  (0.46, 2.32) | 0.98  (0.35, 2.73) | 1.95  (0.43, 8.90) | 0.51  (0.18, 1.43) |
| Highest level of educational attainment – ≤high school/GED (baseline) VS College graduate | 1.03  (0.70, 1.52) | 0.64  (0.24, 1.66) | 1.04  (0.46, 2.38) | 0.71  (0.24, 2.09) | 1.47  (0.33, 6.55) | 0.45  (0.17, 1.23) |
| Highest level of educational attainment – ≤high school/GED (baseline) VS Postgraduate schooling | 0.89  (0.58, 1.36) | 0.89  (0.34, 2.36) | 1.48  (0.62, 3.52) | 0.86  (0.23, 3.22) | 1.31  (0.27, 6.32) | 0.74  (0.26, 2.13) |
| BMI before pregnancy | 1.00  (0.99, 1.02) | 1.06  (1.02, 1.10) | 1.02  (0.99, 1.06) | 0.98  (0.92, 1.05) | 0.99  (0.95, 1.04) | 0.92  (0.87, 0.98) |
| Age at enrollment | 1.00  (0.98, 1.02) | 1.04  (0.98, 1.10) | 1.02  (0.97, 1.06) | 0.97  (0.90, 1.05) | 1.05  (0.98, 1.11) | 0.95  (0.89, 1.01) |
| Birth weight | 1.00  (1.00, 1.00) | 1.00  (1.00, 1.00) | 1.00  (1.00, 1.00) | 1.00  (1.00, 1.00) | 1.00  (1.00, 1.00) | 1.00  (1.00, 1.00) |
| Ever breast fed at 4 months | 1.25  (0.75, 2.08) | 1.53  (0.39, 5.95) | 1.71  (0.50, 5.87) | 0.73  (0.15, 3.49) | 0.58  (0.20, 1.65) | 0.28  (0.11, 0.74) |
| Arsenic in water | 0.99  (0.97, 1.01) | 1.01  (0.98, 1.04) | 1.01  (0.98, 1.04) | 1.00  (0.97, 1.06) | 0.88  (0.77, 1.01) | 1.00  (0.97, 1.03) |

^a^ Outcomes include those treated with prescription medication for upper RTI, lower RTI, acute respiratory symptoms, and fever symptoms and those diagnosed by a doctor for diarrhea and allergy.

^b^ Sample size N = 571 for fever analyses

Supplementary Table S6. Crude Risk Ratio Estimates and 95% Confidence Intervals From GEE in Repeated Measures over 5-18 Months for One Month Earlier Introduction of Rice Cereal on Risk of Immune-related Outcomes, N = 572^a^.

| Outcome | Any report of the outcome  RR (95% CI)  N of total events | Lasting ≥ 2 days  RR (95% CI)  N of total events | Involving a doctor visit  RR (95% CI)  N of total events | Requiring a prescription medication  RR (95% CI)  N of total events |
| --- | --- | --- | --- | --- |
| Upper Respiratory Infections | 1.03  (1.01-1.04)  945 | 1.03  (1.01-1.04)  810 | 1.02  (0.98-1.05)  429 | 1.04  (0.99-1.08)  323 |
| Lower Respiratory Infections | 1.14  (1.01-1.28)  78 | 1.10  (0.97-1.24)  72 | 1.14  (1.00-1.29)  73 | 1.18  (1.01-1.38)  51 |
| Acute Respiratory Symptoms | 1.05  (1.02-1.08)  586 | 1.05  (1.01-1.09)  431 | 1.03  (0.96-1.09)  204 | 1.11  (1.01-1.23)  93 |
| Diarrhea | 1.08  (1.03-1.13)  329 | 1.06  (0.97-1.15)  112 | 0.89  (0.75-1.07)  45 | ^c^  3 |
| Fever Symptoms ^b^ | 1.03  (1.00-1.06)  563 | 1.02  (0.96-1.08)  211 | 1.06  (0.99-1.13)  191 | 1.25  (1.03-1.51)  39 |
| Allergy | 1.17  (1.07-1.29)  91 | N/A ^d^ | 1.19  (1.07-1.33)  60 | N/A^d^ |

Abbreviations: RR, risk ratio; CI, confidence interval.

^a^ Crude GEE. Risk ratios represent increased risk of health outcome with every month earlier of introduction to rice cereal.

^b^ Sample size N = 571 for fever analyses

^c^ Too few observations to perform analysis

^d^ Participants only asked about any allergies and whether these allergies had been doctor diagnosed

Supplementary Table S7. Associations between Rice Cereal Introduction Age in Months and Confounders, N = 572^a^

| Confounder | Estimate | Standard Error | P-Value |
| --- | --- | --- | --- |
| Smoking during any trimester of pregnancy |  |  |  |
| Yes | 0.1970 | 0.1970 | 0.0194 |
| No (reference) |  |  |  |
| Relationship status |  |  |  |
| Married (reference) |  |  |  |
| Single | -0.7455 | 0.2224 | 0.0009 |
| Separated/Divorced | -1.3096 | 0.4715 | 0.0057 |
| Highest level of educational attainment |  |  |  |
| ≤high school/GED (reference) |  |  |  |
| Some college | 0.2373 | 0.2563 | 0.3550 |
| College graduate | 0.4876 | 0.2317 | 0.0359 |
| Postgraduate schooling | 0.7134 | 0.2367 | 0.0027 |
| BMI before pregnancy (kg/m^2^) | -0.0255 | 0.0111 | 0.0223 |
| Age at enrollment (years) | 0.03234 | 0.0132 | 0.0150 |
| Birth weight (g) | -0.0001 | 0.0001 | 0.2810 |
| Ever breast fed at 4 months |  |  |  |
| Yes | 1.1638 | 0.3009 | 0.0001 |
| No (reference) |  |  |  |

^a^ Associations calculated using univariate linear models.
